# The Place of Immunotherapy in the Management of HCV-Induced Vasculitis: An Update

**DOI:** 10.1155/2012/315167

**Published:** 2012-08-15

**Authors:** Laurent Chiche, Stanislas Bataille, Gilles Kaplanski, Noemie Jourde

**Affiliations:** ^1^Department of Internal Medicine, Centre de Compétence pour les Maladies Autoimmunes Systémiques PACA Ouest, Hôpital de la Conception, Marseille, France; ^2^Department of Nephrology, Hôpital de la Conception, Université Aix-Marseille, Marseille, France

## Abstract

Patients with chronic hepatitis C virus (HCV) can develop systemic cryoglobulinemic vasculitis. Combination of pegylated-interferon **α** and ribavirin is the first-line treatment of this condition. However, in case of severe or life-threatening manifestations, absence of a virological response, or autonomized vasculitis, immunotherapy (alone or in addition to the antiviral regimen) is necessary. Rituximab is to date the only biologic with a sufficient level of evidence to support its use in this indication. Several studies have demonstrated that rituximab is highly effective when cryoglobulinaemic vasculitis is refractory to antiviral regimen, that association of rituximab with antiviral regimen may induce a better and faster clinical remission, and, recently, that rituximab is more efficient than traditional immunosuppressive treatments. Some issues with regard to the optimal dose of rituximab or its use as maintenance treatment remain unsolved. Interestingly, in balance with this anti-inflammatory strategy, a recent pilot study reported the significant expansion of circulating regulatory T lymphocytes with concomitant clinical improvement in patients with refractory HCV-induced cryoglobulinaemic vasculitis using low dose of subcutaneous interleukin-2. This paper provides an updated overview on the place of immunotherapy, especially biologics, in the management of HCV-induced cryoglobulinaemic vasculitis.

## 1. Introduction

Chronic hepatitis C virus (HCV) infection is associated with numerous and mostly autoimmune extrahepatic complications. One of the most serious is cryoglobulinaemic vasculitis (CV), which develops in 5–10% of infected patients. CV is a systemic small-vessel vasculitis that affects mostly skin, joints, nerves, and kidneys and can sometimes have a life-threatening presentation [1]. The identification of HCV as the main causal agent for CV has completely modified the management of this virally induced vasculitis. Indeed, circulating immune complexes responsible for organ damage are the result of B-cell expansion and the production of pathogenic IgMs with rheumatoid-factor activity, which is driven by the underlying chronic viral infection. Thus, obtaining a sustained virological response (SVR) has become the main treatment for HCV-induced CV. Fortunately, the combination of pegylated-interferon *α* (peg-IFN-*α*) plus ribavirin has resulted in an SVR in up to two-thirds of patients, depending on the genotype of HCV [2–4]. 

**Figure 1:**
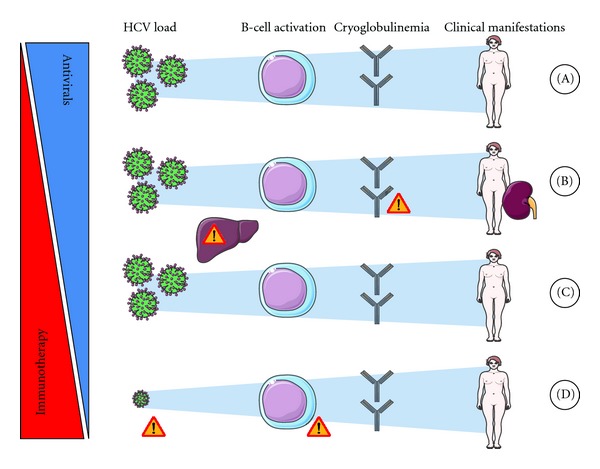
Immunotherapy to manage HCV-induced vasculitis. (A) Antiviral regimen, ideally a combination of interferon plus ribavirin, is the first-line treatment for HCV-induced cryoglobulinaemic vasculitis (CV) when the severity of its manifestations is mild-to-moderate. In addition, short-term low-dose corticotherapy may sometimes be used initially. (B) In cases of severe or life-threatening manifestations (i.e., severe renal involvement), immunotherapy must be initiated immediately. Rituximab has become a preferred choice but, as with other immunosuppressive drugs, careful monitoring of viral load and hepatic functions is necessary. Worsening of vasculitis has been reported in patients just after administration of rituximab, especially in those with serious cryocrit levels, thus, in these patients, corticosteroids and/or plasmapheresis may be initiated before B-cell depletion. An antiviral regimen is initiated either simultaneously or secondarily/sequentially in these patients. (C) When an antiviral regimen is contraindicated, poorly tolerated, or fails to induce a sustained viral remission, immunotherapy is also initiated. Corticosteroids should be avoided when possible. Careful monitoring of viral load/hepatic function is necessary. A prolonged antiviral regimen may be considered when clinical and biological manifestations of MC show an improvement under this regimen in spite of the absence of viral remission. (D) In cases where CV is still active in spite of obtaining a sustained viral response, B-cell malignancy and low-level viremia should be ruled out before considering that the vasculitis is autonomous and before initiating immunotherapy.

However, in some situations, immunotherapy alone or in addition to antiviral treatment is necessary to treat HCV-induced CV (Figure 1). For a long time, immunotherapy for CV has been largely empirical, relying on traditional immunosuppressive options. However, recent studies, including some with a prospectively controlled design, have addressed the place of biologics in this setting. Herein, we aim to provide an updated overview of the place of immunotherapy, especially biologics, for the management of HCV-induced cryoglobulinaemic vasculitis.

## 2. Immunotherapy in HCV-Induced Vasculitis: For Whom and When?

Eradication of HCV with peg-IFN-*α* plus ribavirin is the first-line treatment for CV (Figure 1(a)). Indeed, when this treatment is not contraindicated and sufficiently well tolerated, it allows an SVR in 50% (genotypes 1, 4, 5, 6) to 80% (genotypes 2 and 3) of patients after 48 and 24 weeks of treatment, respectively [2–4]. In these cases, no immunotherapy is needed. However, immunotherapy needs to be considered, alone or in addition to antiviral treatments, in the following situations. 

### 2.1. Severe or Life-Threatening Manifestations

Because of the delayed and uncertain response to antiviral therapy, severe and rapidly progressive CV manifestations (i.e., acute nephrotic or nephritic syndrome, extensive cutaneous ulcers, central nervous system or gastrointestinal manifestations, and hyperviscosity syndrome) require prompt and aggressive treatment (Figure 1(b)). Indeed, the use of aggressive immunotherapy in these settings is indirectly supported by the results of a recent study that identified a strong association between increased mortality and cutaneous ulcers (hazard ratio (HR) 5.37) or renal insufficiency (HR 3.25) [1]. Concerning peripheral neuropathy, even if not considered a life-threatening manifestation, it is a major cause of morbidity in HCV-associated CV and is often refractory to all treatments. In addition, as any improvement is often delayed, later reevaluation prevents a rapid switch to a different therapeutic option if needed, which increases the risk of definitive sequelae. Thus, in the most severe cases, immunotherapy can be a part of first-line treatments [5].

In patients with severe or rapidly progressive manifestations, antiviral therapy is still an important part of treatment and can be initiated either concomitantly or sequentially. Concomitant administration, ideally, may prevent an increase in HCV viral load and hepatic consequences secondary to an immunosuppressive strategy. However, some data support the short-term safety of a sequential strategy (i.e., starting with an immunosuppressive regimen alone), even in patients with advanced liver disease [6]. Also, sequential administration has some practical advantages. First, it avoids situations where the physician faces the occurrence of a side effect within a combined antiviral and immunosuppressive regimen (e.g., cytopenia), a situation that complicates the imputability of this side effect to a specific drug. Also, when renal function is altered, the use of ribavirin is very limited due to increased toxicity. Finally, some authors have reported a paradoxical exacerbation of CV after the initiation of antiviral regimens [7, 8], which may be prevented when immunotherapy is started first. 

### 2.2. Absence of a Virological Response

The use of peg-IFN-*α* combined with ribavirin as the standard-of-care for HCV-induced CV is supported by several studies in which this treatment has been found to be safe and well tolerated and has led to an SVR rate similar to that for HCV-infected patients without CV [9, 10]. But, importantly, only patients with complete clearance of HCV achieve a complete and sustained clinical response, and SVR is not always obtained for various reasons. In about one-third of patients, and particularly those with genotype 1 HCV, a well-conducted antiviral regimen fails [2–4]. In addition, peg-IFN-*α* plus ribavirin is poorly tolerated in 10–20% of patients, leading to early termination of antiviral regimens. Also, some patients have major contraindications to IFN and/or ribavirin, such as advanced age, uncompensated cirrhosis, uncontrolled depressive illness, or untreated thyroid disease. In these patients with CV and no virological response, anti-inflammatory drugs may be warranted to avoid or control severe or debilitating complications (Figure 1(c)). However, a major concern is the potential adverse effects that immunosuppressive therapy could have on the underlying uncontrolled chronic viral infection. Except for severe manifestations (see above), immunotherapy is administered after other therapies have been optimized to obtain an SVR. 

A failed standard-of-care, especially in genotype 1 HCV, may benefit from the recent development of two direct-acting antiviral agents, boceprevir and telaprevir [11]. The combination of one of them to the standard-of-care increases SVR rates in genotype 1 HCV infection to >70%. Alternatively, in virological nonresponders, when a clinical and biological improvement has been observed under an antiviral regimen, some physicians may propose longer treatment for up to 48 or 72 weeks, respectively, for genotypes 2 and 3, and for genotypes 1 and 4 [12]. Also, because of its immunomodulatory properties, interferon may precipitate or exacerbate some preexisting and often subclinical disorders, especially those involving the thyroid, but screening before as well as close monitoring during treatment improves detection and early management of these potential complications [13]. Finally, the contraindications listed above may be judged as relative in some patients, when the benefit of treatment may overcome the theoretical risks. This is especially true for advanced age, but also, in some cases, for depressive status, when antidepressant prophylaxis initiated 2 weeks before interferon therapy may be useful for at-risk patients [14]. 

### 2.3. “Autonomized” Vasculitis

A few patients may experience biological and/or clinical persistence or relapse of CV despite clearance of their HCV infection. This is probably because B-cell expansion has become, at least in part, independent of HCV stimulation (Figure 1(d)). In this setting, underlying B-cell malignancy must be ruled out first. Indeed, HCV-associated CV has been associated with an increased risk of B-cell lymphoma [15]. Landau et al. reported on eight patients who presented with a relapse in HCV-induced CV, despite having achieved SVRs. In two out of three patients whose symptoms of CV persisted and were associated with elevated cryoglobulin levels, B-cell lymphoma was diagnosed [16]. 

There is also controversy about the possible role of occult HCV infection, that is, detectable HCV-RNA in the liver or peripheral blood mononuclear cells in the absence of serum HCV-RNA [17, 18]. Indeed, it is conceivable that the virus, or part of it, may still be triggering B-cell proliferation, although it is not detected in the serum. However, a recent exhaustive review on this topic did not reach any firm conclusions [19]. Recently, we reported, for the first time, the presence of HCV-NS3 viral protein in the kidney of a patient with a similar presentation, but we were unable to conclude on the significance of this finding [20]. What is certain for now, is that an ultrasensitive real-time PCR assay should be conducted on the serum and/or cryoprecipitate to rule out low-level infection, which may have been misdiagnosed as occult infection in previous studies [21]. Thus, in patients with an SVR but persistent clinical manifestations of CV, after exclusion of underlying hemopathies and/or low-level HCV-persistent infections, the autoimmune component of the disease may be considered as autonomized and treated similarly to nonvirally related CV [22].

## 3. Immunotherapy in HCV-Induced Vasculitis: Which One?

Various anti-inflammatory drugs that are used successfully to treat other types of vasculitis are also used to treat HCV-induced vasculitis. However, during the last decade, conventional immunosuppressive treatments (i.e., cyclophosphamide and plasmapheresis) have been progressively challenged by biologics. Indeed, the most common cause of death in patients with CV is infection and, in the study of Landau et al. [1], immunosuppressive treatment was associated with an increased risk of death, independently of disease severity (HR 6.51), suggesting that a more targeted immune-based strategy would be beneficial. Apart from the poor effectiveness of TNF-blockade by infliximab or etanercept, reported by us and others [23–25], or the recent anecdotal report of the successful use of an anti-interleukin(IL)-6 strategy [26], rituximab (RTX) is, to date, the only biologic that has sufficient evidence to support its use for this indication. Interestingly, to balance this anti-inflammatory strategy, a recent pilot study reported the success of a proregulatory strategy with low-dose IL-2 [27].

### 3.1. Anti-Inflammatory Strategy: Rituximab

RTX is a monoclonal antibody against the CD20 antigen, which is selectively expressed on B cells. The rationale underlying RTX treatment is that in CV, CD20-positive cells are expanded, activated, and play a pivotal role in cryoglobulin production [28]. Several studies have demonstrated that RTX is highly effective when CV is refractory to antiviral regimens [5, 6, 29–31], that the association of RTX with an antiviral regimen may induce a better and faster clinical remission [32, 33] and, recently, that RTX is more efficient than traditional immunosuppressive treatments [34, 35].

With some variations according to the different manifestations of CV, the overall response rate to rituximab in patients refractory to antivirals has been reported in recent meta-analyses to be ≥80% [36, 37]. The delay in response is variable, but improvement occurs within 1–6 months. Recent studies that have compared a combined therapy with RTX to antiviral therapy alone show that a combined therapy may be the best choice for patients with severe manifestations of CV. Indeed, in a prospective cohort study of 93 patients, combined therapy reduced the time to clinical remission and improved renal-response rates compared to peg-IFN-*α*  + ribavirin alone [33]. In another prospective study that included 37 patients, those in the RTX group achieved a complete response more often than patients not receiving RTX (54.5% versus 33.3%) [32].

The rationale for choosing a targeted therapy with RTX instead of conventional immunosuppressive agents has been only poorly supported by evidence, though two recently published studies have filled this gap [34, 35] (Table 1). The first study [34], an open-label randomized controlled trial (RCT) conducted in Italy, compared RTX to conventional therapies (i.e., corticosteroids, plasmapheresis, azathioprine, or cyclophosphamide) in 57 patients with severe manifestations of CV. Of note, patients in the conventional-therapy group, whose treatment failed, had the opportunity to crossover and receive RTX. At 12 months, the proportion of patients who continued their initial therapy was significantly higher in the RTX group, and only 13.8% of patients in the conventional-therapy group continued their initially assigned therapy beyond 3 months. The second study [35], conducted in the US, was also an open-label RCT, which compared RTX and standard therapy in 24 patients with HCV-related CV. Standard therapy was considered to be maintenance or intensification of conventional immunosuppressive therapy, but the patients receiving RTX were allowed to continue their background immunosuppressive therapy. At 6 months, clinical efficacy was clearly greater for RTX compared to conventional immunosuppressive therapy. Thus, even though the design of these studies may have advantaged RTX (Table 1), the data support a preference for targeted B-cell depletion with RTX as the agent of choice for CV. They also provide additional information on the modalities of administration of RTX and its safety.

Indeed, as in other autoimmune conditions [38], there is no consensus on the choice of the modality of administration, that is, a “rheumatological” regimen: 4 weekly infusions of 375 mg/m^2^ versus a “hematologic” regimen: 2 biweekly infusions of 1000 mg, which are equally used in practice as well as in RCT (Table 1). However, Sène et al. have raised the issue of serum sickness following the use of RTX therapy for CV, especially in patients with the highest cryoglobulin levels and the lowest C4 levels [39]. RTX may form a complex with cryoglobulin, which could increase cryoprecipitation and induce severe systemic reactions, including serum sickness. Consequently, these authors propose the use of a lower starting dose of RTX (i.e., rheumatological regimen), possibly preceded by corticosteroids and/or plasmapheresis to avoid side effects. Overall, short-term reactions to RTX infusions do not seem to be more frequent in CV than in other autoimmune conditions that are treated with a classical premedication of 100 mg of methylprednisolone, antihistamine drugs, and paracetamol.

The safety of RTX, especially when RTX is used without the cover of antiviral agents, was supported in both RCTs, even though HCV load was not monitored in the Italian study [34]. RTX was not associated with significant liver impairment despite transient increases in HCV viral load, as already reported when RTX was given to patients with liver cirrhosis [6]. Nevertheless, data on HCV load and liver enzymes come from small sample-sized studies [40] with short-term followups, thus, this needs further evidence. RTX is also associated with a significant risk of infection, especially in patients with renal failure and advanced age and in those receiving high doses of corticosteroids [41]. This warrants the same precautions recommended for other autoimmune conditions with regards to vaccination and specific followup [42], including also early identification of rare but potentially severe complications related to RTX (i.e., anti-Pr cold agglutinins syndrome or progressive multifocal leukoencephalopathy). 

### 3.2. Proregulatory Strategy: IL-2

Recently, Saadoun et al. obtained significant expansion of circulating regulatory T lymphocytes (Treg) with concomitant clinical improvement in 8/10 patients with refractory HCV-induced CV using a low dose of subcutaneous IL-2 (Proleukin, 1.5 million IU per day for 5 days, then 3 million IU per day for weeks 3, 6, and 9) [27]. Their patients were refractory to previous antiviral regimens, but only 1/10 patients had previously received rituximab, and only 1/10 had mild renal involvement. Interestingly, these patients did not receive any corticosteroids during the study period. The limitations of this pilot study are the absence of a control group, the short follow-up time (a few months), and some potential confounding factors (i.e., there was also a significant increase of CD56 bright NK cells), which prevent definitively concluding that the clinical benefits were solely due to the observed increase in Treg cells. Indeed, in the study by Koreth et al. (published at the same time), and also using low-dose IL-2 in patients suffering from graft-versus-host-disease, Treg-cell counts increased in all patients but were not statistically different between patients who had and those who did not have a response [43]. Nevertheless, these two studies constitute a proof of principle that low-dose IL-2 can be used safely to promote tolerance, probably through Treg expansion [27, 43]. 

IL-2 is produced by naive and memory T cells after antigen stimulation and binds to a high-affinity receptor consisting of three subunits: IL-2R*α* (CD25), IL-2R*β* (CD122), and *γ*c (CD132). Until recently, almost all clinical trials using IL-2 aimed at boosting effector T lymphocyte (Teff) function and have taken advantage of the immune-stimulating activity of IL-2. Indeed, this approach was successful in a subset of patients suffering from renal cell carcinoma and melanoma [44] and was also tested to boost the immunity of patients with AIDS [45]. The main limitations to the broader use of IL-2 are its very short half-life in the circulation after infusion, which necessitates using IL-2 at levels as high as possible, and its life-threatening nonspecific toxicities, such as vascular-leakage syndrome. Recent studies have shown that the primary function of IL-2 is, actually, the generation and survival of Treg [46], which explains in part why this approach failed in its anticancer indication and supports the possibility that IL-2 may, instead, promote T-cell tolerance in autoimmune conditions, such as CV, where a deficit of Treg has been documented [47]. 

**Figure 2:**
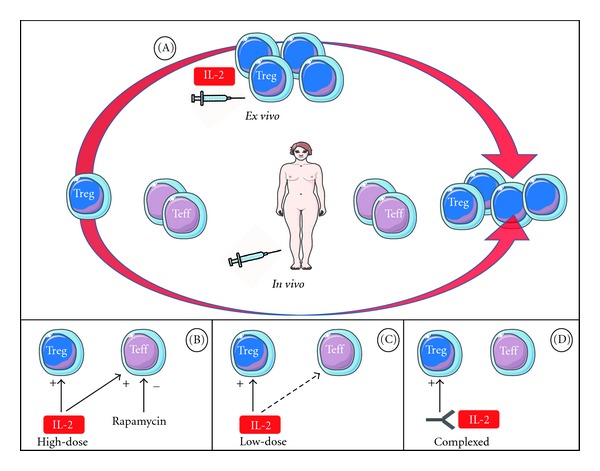
Different IL-2 based approaches to promote the expansion of regulatory T cells (Treg). A first approach consists of using IL-2, together with other stimuli, to expand *ex vivo* the Treg cells collected from a patient's tissue culture before transferring these cells to the patient (A). *In vivo*, IL-2 can be administered subcutaneously at high doses but can be associated with rapamycin to prevent activation of effector T cells (Teff) (B) or given at a low dose for the same reason (C). IL-2-specific monoclonal antibodies can be used to target IL-2 selectively to Treg cells (D).

There are several ways to use IL-2 to boost Treg. IL-2, together with other stimuli, can be used to expand the Treg-cell population *ex vivo* (Figure 2(a)), in tissue culture, before transferring these expanded cells to patients [48]. But this strategy is probably too complex to broadly translate to the bedside. Conversely, *in*-*vivo* expansion using subcutaneous infusion of IL-2 has been already used with variable results in mice and humans. IL-2 can be used at a high dose with coadministration of rapamycin to prevent the activation of Teff cells without affecting the Treg-cell response (Figure 2(b)). This protocol has proved to be beneficial in the treatment of diabetes in NOD mice [49] but, unfortunately, a clinical trial in new-onset type-1 diabetes patients showed that treatment with rapamycin plus a relatively high-dose of IL-2 (4.5 × 106 IU/day subcutaneously, three times a week for 4 weeks) resulted in greater loss of insulin secretion at 3 months and, overall, was considered to worsen pancreatic *β*-cell function [50]. A low dose of IL-2 alone may also be used, which favours the expansion of Treg and has only a minor effect on Teff (Figure 2(c)). This strategy was successful in the two clinical studies already mentioned [27, 43] but warrants confirmation on a larger scale and additional work is needed to fully understand the role of IL-2 on cells other than Treg. Finally, an alternative approach (Figure 2(d)) could be the use of improved IL-2 formulations or IL-2-specific monoclonal antibodies, which allow IL-2 to selectively target Treg cells [51].

## 4. Biologics in HCV-Induced Vasculitis: Next Steps

In just a few years, biologics have modified the management of HCV-related CV. Their use has also raised many unsolved issues. The first concerns maintenance treatment. In patients refractory to antiviral regimens and who are successfully treated with RTX, more than a third will relapse during B-cell recovery, usually between 6 and 12 months [36, 37]. However, retreatment with RTX after a relapse seems to be effective in most cases [34, 35]. Systematic maintenance of RTX therapy has rarely been reported in CV but may be considered in severe forms [52], though the best modality remains to be determined. Other biologics targeting B cells, such as other anti-CD20 monoclonal (i.e., ocrelizumab and ofatumumab), anti-CD22 (epratuzumab), or anti-BAFF (belimumab) might also prove useful in the management of these conditions.

The second concern is the dosage used in RTX regimens. As already stated, both “haematological” and “rheumatological” regimens are both used in practice. Visentini et al. recently reported preliminary results from 27 patients receiving low-dose rituximab (2 weekly doses of 250 mg/m^2^): they had a response rate similar to that reported for patients treated with standard doses [53]. If confirmed, this regimen could reduce costs, improve safety profiles, and be preferred by patients with nonsevere manifestations. Finally, one additional advantage of using RTX to treat CV may be the reduced exposure to corticosteroids. In the two RCTs [34, 35], responders to RTX therapy received lower total doses of prednisone than those allocated to a conventional immunosuppressive therapy. The possibility to propose a steroid-free regimen in selected patients with CV and to be only treated with RTX warrants additional trials. 

In conclusion, patients suffering from HCV-induced vasculitis have and will largely benefit from the progress made in both antiviral and immunologic research. It seems that the place of biologics in the management of this complex condition is likely to increase in a near future.

## Figures and Tables

**Table 1:** Prospective randomized controlled trials comparing rituximab (R) with a classical immunosuppressive regimen (C).

Studies	Sneller et al. (USA)	De Vita et al. (Italy)
Methodology		

Sample size (R/C)	24 (12/12)	57 (29/28)
Design	Prospective RCT Open-label, monocentric	Prospective RCT Open-label, multicentric
Followup duration	M12	M24
Rituximab	375 mg/m^2^ × 4 No GC premedication	1000 mg × 2 100 mg MP iv before each
Other treatments allowed for group R	IS/GC already initiated	Low dosage of GC
Effective regimen for group C	IS/GC already initiated ± increase (only PL = 1 at M5)	GC = 17 or IS = 7 (AZA/CYC) or PL = 5 ± GC
Planned sample size	30	124
Limitations	8-year enrolment Early stop after interim analysis	86% switch before M2^∗^ Early stop after interim analysis

Patients		

Underlying VHC infection	24/24	53/57
Previous treatments (R versus C)	Unbalanced at randomization GC = 6 versus 3 CYC = 1 versus 0, PL = 2 versus 0	Not provided

Efficacy		

Primary endpoint	Clinical remission at M6	Survival of initial treatment at M12
Result (R versus C)	10/12 (83%) versus 1/12 (8%)	64% versus 3.5%
Response to retreatment	R: 3/3	R: 5/7 C: 6/8
Time of switch of C to R	After M6	As soon as failure^∗^
Number of switches of C to R	9/12	23/28
Response to switch to R	4/7 (2 lost to followup)	14/23

Safety		

Infusion-related severe events	1 serum-infusion reaction	1 hypotension with angina
Viral load of VHC	No difference	Not monitored

Abbreviations: AZA: azathioprine; CYC: cyclophosphamide; GC: glucocorticoids; IS: immunosuppressive; MP: methylprednisolone; PL: plasmapheresis.
